# Needle-Based Electrical Impedance Imaging Technology for Needle Navigation

**DOI:** 10.3390/bioengineering10050590

**Published:** 2023-05-13

**Authors:** Jan Liu, Ömer Atmaca, Peter Paul Pott

**Affiliations:** 1 Institute of Medical Device Technology, University of Stuttgart, 70569 Stuttgart, Germany; oemer.atmaca@ito.uni-stuttgart.de (Ö.A.); peter.pott@imt.uni-stuttgart.de (P.P.P.); 2 Institute of Applied Optics, University of Stuttgart, 70569 Stuttgart, Germany

**Keywords:** bioimpedance, electrical impedance imaging, impedance measurements, needle navigation, tissue classification

## Abstract

Needle insertion is a common procedure in modern healthcare practices, such as blood sampling, tissue biopsy, and cancer treatment. Various guidance systems have been developed to reduce the risk of incorrect needle positioning. While ultrasound imaging is considered the gold standard, it has limitations such as a lack of spatial resolution and subjective interpretation of 2D images. As an alternative to conventional imaging techniques, we have developed a needle-based electrical impedance imaging system. The system involves the classification of different tissue types using impedance measurements taken with a modified needle and the visualization in a MATLAB Graphical User Interface (GUI) based on the spatial sensitivity distribution of the needle. The needle was equipped with 12 stainless steel wire electrodes, and the sensitive volumes were determined using Finite Element Method (FEM) simulation. A k-Nearest Neighbors (k-NN) algorithm was used to classify different types of tissue phantoms with an average success rate of 70.56% for individual tissue phantoms. The results showed that the classification of the fat tissue phantom was the most successful (60 out of 60 attempts correct), while the success rate decreased for layered tissue structures. The measurement can be controlled in the GUI, and the identified tissues around the needle are displayed in 3D. The average latency between measurement and visualization was 112.1 ms. This work demonstrates the feasibility of using needle-based electrical impedance imaging as an alternative to conventional imaging techniques. Further improvements to the hardware and the algorithm as well as usability testing are required to evaluate the effectiveness of the needle navigation system.

## 1. Introduction

Current healthcare practices often require needle insertions through tissue layers such that the needle tip is positioned in a particular region of interest. These procedures involve blood draw, medication delivery, tissue biopsies, and cancer treatments such as brachytherapy, radiofrequency ablation, and cryoablation. For example, 70% of patients in acute care settings require a Peripheral Intravenous Catheter (PIVC) [1]. Worldwide, over one billion PIVCs are placed in hospitalized patients annually [2]. Despite the high frequency of interventions, complications still arise fairly often due to incorrect needle positioning [3,4]. Minor bruising and hematoma are the most frequent complications observed [5]. Other consequences can include pain, sclerosis, phlebitis, vein occlusion, nerve punctures, and infections. In many cases, the puncture has to be repeated, which jeopardizes the safety and well-being of the patient [6]. The errors arise from uncertainties that the intervention entails. At present, venipuncture is carried out by hand after the target blood vessel has been located and the appropriate angle and depth of insertion have been determined. The only indication of the correct depth of insertion is a change in the force required to push the needle forward or the appearance of a blood “flash” in a designated window of the needle. As a result, the success rate is heavily influenced by the expertise of the clinician and the patient’s physiology [3,4].

In terms of needle guidance, ultrasound imaging can be seen as the gold standard. Ultrasound requires minimal preparation, does not involve ionizing radiation, and provides continuous information about the needle position. However, it is not ideal as the spatial resolution is deficient [7]. In general, ultrasound images tend to be noisy due to reflections, reverberations, shadows, air pockets, and biological speckle, resulting in a possible incoherence of the reconstructed image of the distal end from the echo and the actual tip of the needle. Lastly, the interpretation of the 2D images is highly subjective, and slight needle movements are often required to avoid misjudging of the images [8,9].

Another possibility for needle guidance that is being researched is the identification of tissue types by means of bioimpedance measurements. This approach is based on the variance of dielectric properties of different tissue types. Research around needle impedance measurements includes the optimization of electrode geometries in order to achieve the best possible spatial resolution, which corresponds to the smallest possible sensitivity field. The smaller the sensitive volume, the more accurate the tissue information provided. As the spatial sensitivity distribution cannot be directly determined experimentally, Finite Element Method (FEM) simulation has proven to be a useful method [10,11,12].

It has been shown that monopolar measurements, which are characterized by a high current density close to the active electrode surface, can be obtained by using a sufficiently large neutral electrode in a two-electrode setup [13,14]. The volume sensitivity, which is a function of the squared current density in a given tissue volume, was found to be small enough to neglect the area outside the sensitive area. Thus, the impedance is dominated by the tissue in the vicinity of the active electrode. Monopolar setups were used to investigate the discrimination of muscle and fat for drug administration [15,16], the detection of venous blood [17], and intraneural injections [18].

Bipolar setups are characterized by the utilization of two similar electrodes for both carrying the current and picking up the voltage. Various simulative investigations were conducted showing different needle geometries that minimize the sensitivity field [11,12,19,20]. Bipolar setups exist, which use at least one needle body as active electrode or even two nested needles [12,20,21,22,23]. In other works, two active electrodes were installed at the needle tip [24,25,26,27,28], or a concentric needle electrode designed for Electromyography (EMG) was used [29].

Tetrapolar setups can improve the accuracy of a measurement by using four electrodes: two Current-Carrying (CC) and two (voltage) Pick-Up (PU) electrodes. In this way, the voltage at the PU electrodes is less influenced by the polarization effects at the CC electrodes. Current density distributions for tetrapolar or even pentapolar setups have been studied [30,31]. The integration of tetrapolar electrode setups was studied by means of wire electrodes made of stainless steel [30] and flexible thin-film sensors [32]. Efforts have also been made to increase the extent of information that impedance measurement setups can provide. Therefore, the integration of multiple electrode setups on a single needle has been proposed, which we refer to as multi-local impedance measurements [30,32,33]. The aim is to provide localized impedance information at different locations at the needle [33].

In this work, we suggest that localized impedance information can be exploited to realize an imaging technology that can be used for needle navigation. Our proposed concept of needle-based electrical impedance imaging involves acquiring tissue information at multiple locations along the needle and defining the boundaries of the identified tissue types. By combining individual tissue information, we can generate a 3D visualization. To demonstrate the feasibility of our concept, we developed an electronic system capable of measuring impedance and switching between electrode pairs. Additionally, we modified standard hypodermic needles by incorporating 12 electrodes. To interpret the impedance data, we conducted an extensive analysis of the sensitivity distribution of our needle through FEM simulation. For tissue identification, we employed a Machine-Learning (ML) approach based on recorded data for training. Finally, we integrated all the data and visualized it in a 3D Graphical User Interface (GUI), which serves as a tool for needle navigation.

## 2. Materials and Methods

### 2.1. Sensor Development

**Figure 1:**
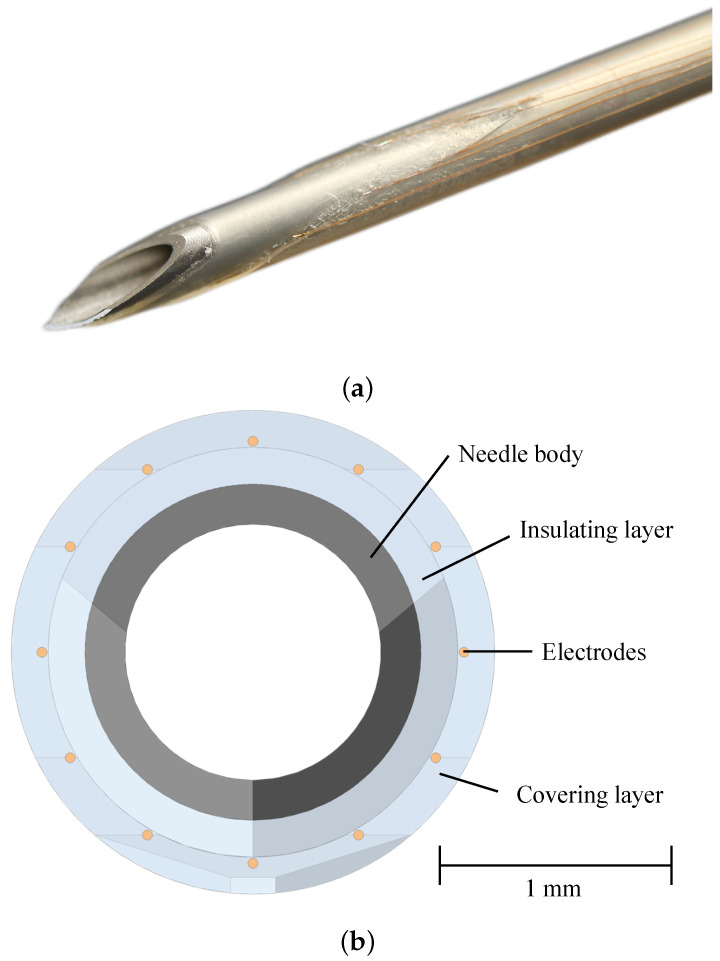
Design of a 14 Gauge (G) hypodermic needle equipped with stainless steel wire electrodes. (**a**) Picture of a sensor-integrated hypodermic needle. (**b**) Front view of the Computer-Aided Design (CAD) needle model.

The development process of the sensing electrodes was derived from the main requirements we set for this research project. To preserve the function as a hollow needle, the electrodes had to be mounted on the outside of the needle. We adopted a previously reported fabrication protocol and altered it slightly [30]: a 14 Gauge (G) hypodermic needle (Sterican, B. Braun SE, Melsungen, Germany) with an outer diameter of 2.1 mm served as a basis. As a first step, a Polyvinylidene Difluoride (PVDF) heat shrink tube (DERAY-KY 175, MCE Mauritz Electronics, Nieder-Olm, Germany) with an inner diameter of 3.2 mm and a shrink ratio of 2:1 was applied to insulate the needle. We used a scalpel to cut the protruding piece of the heat shrink tube to match the shape of the needle tip. Afterwards, the surface of the heat shrink tube was evenly coated with a sprayable adhesive (3M Repositionable 75 Scotch-Weld, 3M Deutschland GmbH, Neuss, Germany). Next, 12 stainless steel wire electrodes (AISI 316L) with a circular cross-section with a diameter of 0.06 mm (Zivipf.de, Thomas Schmoll, Treuchtlingen, Germany) were placed circularly around the needle tip, each 30° apart and displaced by 2 mm in the axial direction (cf. Figure 1). The wire endings were galvanized with gold. The second layer of the heat shrink tube was applied afterwards, and the excessive piece was cut so that 1 mm of the wire endings was exposed. The remaining adhesive was removed eventually. The proximal part of a needle that has been equipped with electrodes in this way can be seen in Figure 1.

**Figure 2:**
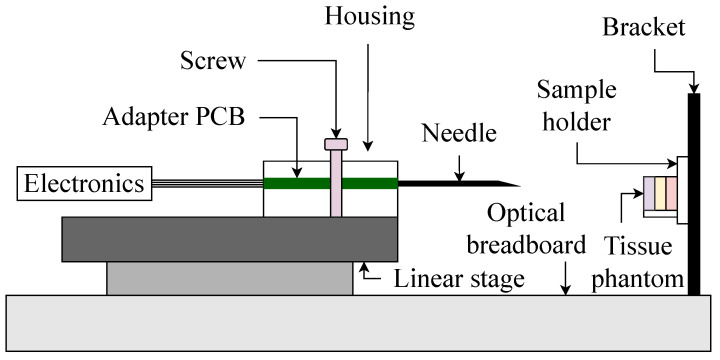
Schematic representation of the experimental setup.

The galvanized wire ends at the distal part were contacted electrically to an adapter Printed Circuit Board (PCB). To connect the needle to the PCB mechanically, we 3D printed a male Luer–Slip connector attached to the PCB. The PCB with the needle is placed in a small housing mounted to a motorized linear stage (LINAX Lcx 80F40, Jenny Science AG, Rain, Switzerland) that is providing the movement (cf. Figure 2). A servocontroller (XENAX Xvi 75V8, Jenny Science AG) provides the motor control.

### 2.2. Experimental Design

**Figure 3:**
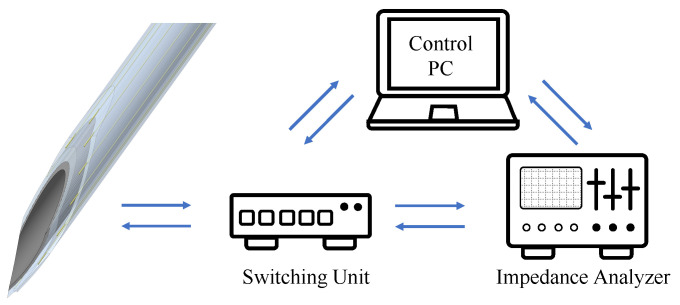
Overview of the components used for the needle navigation system.

The measurement system for the needle navigation consists of an Impedance Analyzer (IA, Sciospec ISX-3v2, Sciospec Scientific Instruments GmbH, Bennewitz, Germany) and a switching unit (cf. Figure 3). For an unobstructed workflow and a low latency of the needle navigation system, we use a single measurement frequency rather than an impedance spectrum. The IA is constantly measuring with an excitation voltage of 200 mV and a frequency of 100 kHz. The switching unit is responsible for switching the different electrode pairs. It comprises a microcontroller (Arduino Nano V3.0, Arduino LLC, Somerville, MA, USA) and two analog 16:1 multiplexer (CD74HC4067, Texas Instruments Inc., Dallas, TX, USA). Both systems are connected to a control PC (i5-9500, 32 GB RAM, Intel UHD Graphics 630). We developed a GUI in MATLAB (MATLAB R2022a, The MathWorks Inc., Natick, MA, USA) that allows the control of the measurement.

For our experiments, we used tissue phantoms with realistic electrical properties. In the case of venipuncture, we fabricated phantoms that mimicked blood, fat, and dermis (skin). The phantoms are based on a mixture of water, gelatine, and agar. In this way, the phantoms provide enough mechanical stability and the possibility to use molds for shaping. The electrical properties are adjusted by adding sodium chloride and propylene glycol. The ingredients for phantom fabrication can be seen in Table 1 [22,34].

For training the classification algorithm, we recorded impedance measurement data using the modified needles. For this purpose, we inserted a needle into the tissue phantom and captured the measured impedance values for each excitation state. As there are 12 different excitation states, 12 measurements are taken each run. We repeated the procedure for three needles and collected a total of 420 impedance values for each tissue type.

We conducted two different tests to demonstrate the sensing capability of the needle and to evaluate the classification algorithm. The first test included the manual insertion of the needle into individual tissue types. The impedance data for each excitation state were recorded and classified according to the classification algorithm. This procedure was repeated five times for each tissue phantom type. In the second test, we used the linear stage to perform a controlled needle movement into a layered phantom with different tissue types to verify the classification and the ability to determine the puncture depths. Each tissue phantom had a thickness of 1.5 cm in the sample holder. The distance from the foremost electrode to the rearmost electrode in the longitudinal direction was approximately 1.2 cm. The linear stage inserted the needle with 1 mm/s and with position increments of 3 mm. There were therefore two positions in which the needle part covered with electrodes is located completely in one type of tissue phantom. To assess the classification success rate, we only evaluated the impedance data at positions with complete coverage by one type of tissue phantom. Three insertions with three different needles were performed. Each insertion involved two positions in three different tissue types. In total, there were 54 positions that are evaluated. Each position yielded 12 different excitation states, resulting in 648 classifications altogether. The puncture depth could be derived from the combination of absolute position given by the linear stage and the measured impedances at the known electrode positions along the needle. To verify the position of the needle tip, we used pictures of the needle taken from the top view.

### 2.3. Simulation

The simulative analysis by means of FEM is pivotal for the imaging method. It determines the spatial sensitivity distribution. The simulations were performed with COMSOL Multiphysics version 5.6 (Comsol Multiphysics GmbH, Göttingen, Germany). We used the Alternating Current/Direct Current (AC/DC) and the Computer-Aided Design (CAD) Import module for the simulations. All simulations were conducted on a simulation PC (i9 12900K, 96 GB RAM, RTX3080 Ti).

#### 2.3.1. Geometry

We defined a cube with a side length of 30 mm, which represents a piece of homogeneous and isotropic tissue. The tissue type was changed in between the simulations. After that, we imported the CAD model of the modified needle. Both parts were connected using the form union node to constitute combined geometric domains.

#### 2.3.2. Material Properties

Electrical conductivity and permittivity are the most important properties for our purposes. The parameters that we used are derived from the literature and can be found in Table 2 [34,35,36,37,38].

#### 2.3.3. Boundary Conditions

We set the boundary conditions using the Electric Current (EC) interface. We simulated a bipolar measurement with a terminal source of 1 A (unity current) assigned to the excitation electrode and a ground terminal assigned to the counter electrode. Electrical isolation boundaries were inserted between all interfaces except for the interfaces between the tissue and the measuring electrodes. This ensured that the current flowed exclusively between the measuring electrodes without penetrating into other materials. These simulation settings were implemented in eleven other EC interfaces to simulate different excitation states with different electrode pairs.

#### 2.3.4. Meshing

As a next step, a suitable mesh had to be found to divide the geometry into finite elements. Starting from a very coarse mesh, the mesh was modified until a compromise between mesh resolution and computational effort was found. The final mesh consisted of four separate meshes. The first mesh was created by assigning a two-dimensional Free Triangular mesh to the surface boundaries of the outward wire ends. The surface boundaries of the outward end of the needle as well as the surface boundaries of the two insulation layers were also equipped with a Free Triangular mesh. To extend the two-dimensional mesh into the third dimension, a Swept Mesh was set in such a way that for each surface element, a long prismatic element is created, which extends from the outward end of the needle to the tissue surface. The second mesh comprised the wire tips, which are constructed as Free Tetrahedral mesh. In addition, the third mesh for the tissue and the fourth mesh for the needle tip including the insulation were meshed as Free Tetrahedral with slightly different resolutions.

#### 2.3.5. Post-Processing

The transfer impedance between a CC electrode pair and a PU electrode pair is given by

(1) $$Z_{\mathrm{tr}}=\;\int \int \int \;\rho J_{\mathrm{CC}}^{{\;}^{\prime }}\cdot J_{\mathrm{reci}}^{{\;}^{\prime }}dV$$

where ρ is the resistivity of the medium, $J_{\mathrm{CC}}^{{\;}^{\prime }}$ is the normalized current density vector field from a unit current applied to the CC electrodes, and $J_{\mathrm{reci}}^{{\;}^{\prime }}$ is the normalized current density from a unit reciprocal current applied to the PU electrodes [39]. The sensitivity of a tissue voxel is defined as

(2) $$S=J_{\mathrm{CC}}^{{\;}^{\prime }}\cdot J_{\mathrm{reci}}^{{\;}^{\prime }}.$$

In a two-electrode system (bipolar or monopolar), the forward and reciprocal current densities are identical $\left(J_{\mathrm{CC}}^{{\;}^{\prime }}=J_{\mathrm{reci}}^{{\;}^{\prime }}=J^{{\;}^{\prime }}\right)$. Therefore, $S=|J^{{\;}^{\prime }}{|}^{2}$. The bulk of the sensitivity, which we refer to as sensitive volume, defined as 97% of its accessible value range, is in the direct vicinity of the electrodes [10].

Due to simulation singularities, we used the line method to handle unphysical behavior [40,41]. The method involved constructing a geometric line starting from the singularity and pointing into the free tissue space. Sensitivities were evaluated along this line for a finer and a coarser mesh. By comparing the relative differences in sensitivity with respect to the original mesh, we could define an acceptable error margin and limit the sensitivity to the maximum value found at a certain distance away from the singular point.

### 2.4. Software Architecture

The control software comprised control modules for the switching circuitry, the IA, and the linear stage. In addition, the tissue classification algorithm was implemented as well as the 3D visualization. The main requirements for the software were a low latency between the actual measurement and the representation in the visualization as well as the possibility of displaying the tissue around the needle in three dimensions. We aimed for a scanning frequency of the electrodes in the range of 7–30 Hz.

The switching circuitry had two functions: switching of the multiplexers and providing the information about the current switching state. Both functions were implemented in an Arduino Integrated Development Environment (IDE) script. The data stream of the Arduino was read by the MATLAB GUI. The IA was controlled via the Communication Port (COM) interface. The linear stage was connected through a Transmission Control Protocol/Internet Protocol (TCP/IP) connection via an ethernet cable. The motor controller used an American Standard Code for Information Interchange (ASCII) protocol to control the motor movement. We developed MATLAB libraries that translate all commands into MATLAB functions.

For tissue classification, we implemented an ML-based algorithm. We defined a class for data preparation that conditions the simulated impedance data in an interpretable way. In addition, needle models as well as sensitivity fields were loaded. We chose a k-Nearest Neighbors (k-NN) algorithm with k=34 neighbors and an Euclidean norm as classifier; i.e., the 34 data points closest to the query point, the 34 nearest neighbors, were determined. Since every neighbor was labeled, the query point was assigned the class that was represented most frequently among the 34 neighbors.

The visualization was the central interface for the user. As we considered both manual insertions as well as automated insertions, a local and a global visualization mode were implemented. The visualization concept involves the utilization of the needle CAD model as a basis. Depending on the classified tissue type for a particular electrode pair exciting, the sensitive volume around the electrode pair is displayed in a certain color, which is representative for the identified tissue type.

For local visualization, i.e., manual insertion, we had no feedback about the orientation and position of the needle. Only the local circumstances (classified tissue types) surrounding the needle were displayed. For the global visualization, i.e., automated insertion, we could use the position feedback from the linear stage to display the needle inside a coordinate system. Assuming the tissue was relatively stiff with little deformation, we could assign particular coordinates to the classified tissue types and display them. The sensitive volumes were represented by isosurfaces enclosing all tissue voxels that accounted for up to 97% of the total sensitivity.

The latency was determined using MATLAB’s tic/toc commands. A measurement was finished as soon as a callback was triggered by the Arduino’s excitation state change. The tic command registered the starting time for data processing, classification and visualization, and the toc command recorded the end time.

### 2.5. Needle Navigation Software

**Figure 4:**
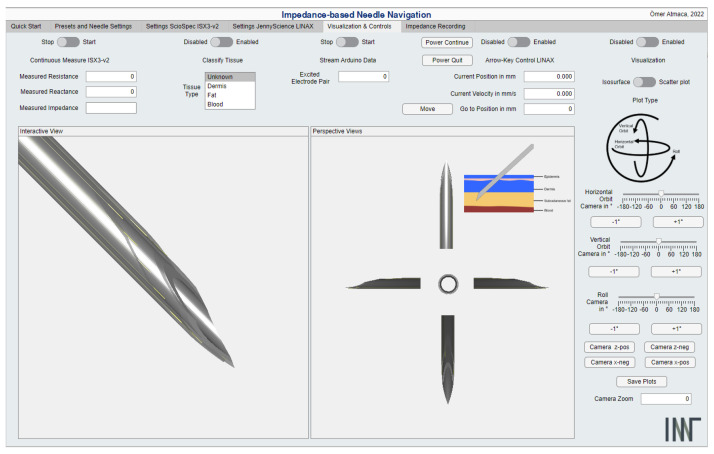
MATLAB Graphical User Interface (GUI) for needle navigation. The GUI provides control options for the measurement, the visualization area, and the control buttons for the visualization.

**Figure 5:**
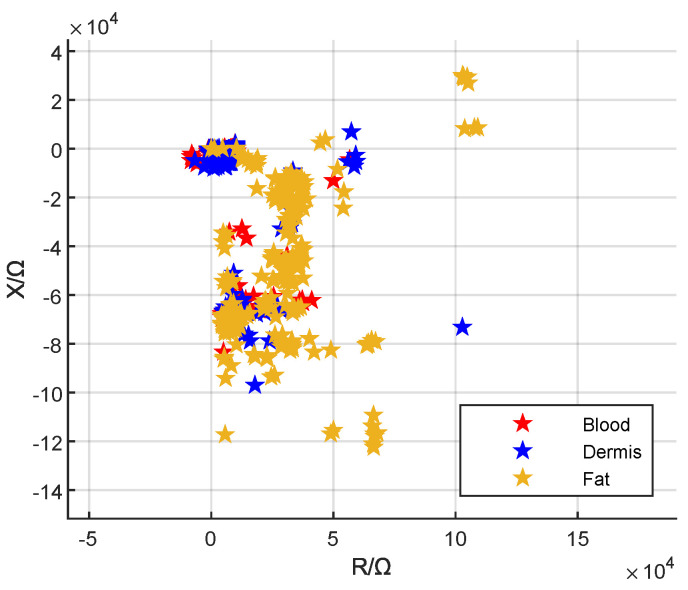
Measured impedance data depicted in the complex number plane.

The IA and linear stage manufacturer’s software packages were used to create a GUI that provides an intuitive interface for measurement, control, and visualization. Figure 4 shows the visualization tab of the MATLAB GUI. Further tabs allow the control and the adjustment of measurement settings. The measured data which were recorded for the k-NN algorithm can be seen in Figure 5.

## 3. Results

**Figure 6:**
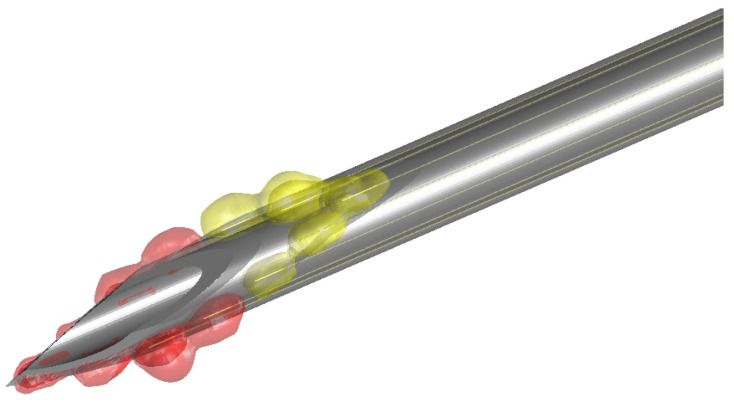
Exemplary needle visualization. The needle is surrounded by color-coded volumes. The sizes of the sensitive volumes have been predetermined by simulation. In this scenario, the needle tip has just reached blood, which is indicated by the red volumes. The more distal electrodes have identified fat. The volumes are colored yellow.

**Figure 7:**
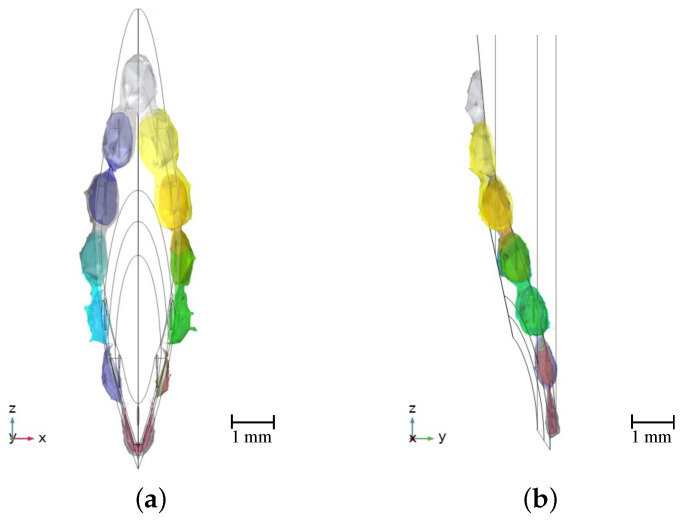
Normalized sensitivity distributions (%) of the needle model for a frequency of 100 kHz. The colored isosurfaces represent the outer boundaries of the sensitive volumes. (**a**) Front view. (**b**) Side view.

Figure 6 demonstrates the 3D visualization of different tissue types around the needle. Venipuncture serves as an exemplary application here. Red volumes represent blood, and yellow-colored volumes depict fat tissue. Figure 7 shows the sensitive volumes around the electrodes, which were obtained by simulation.

The average latency between measurement and visualization was 112.1 ms (standard deviation 66.4 ms) and the median was 86.9 ms (interquartile range 40.1 ms).

### 3.1. Local Visualization

**Figure 8:**
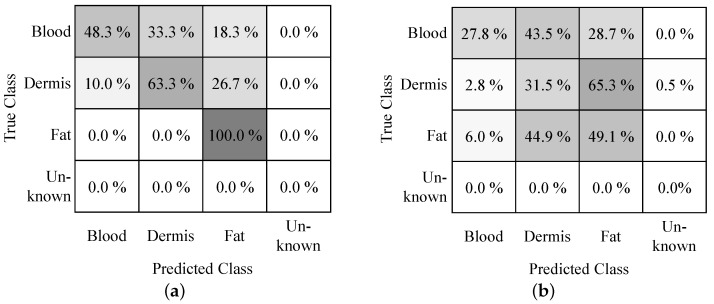
Classification success rates depicted in confusion matrices. (**a**) Local visualization. (**b**) Global visualization.

The classification results are summarized in Figure 8. For the blood phantom, the overall success rate of the classification is 48.33%; i.e., 29 out of 60 classification attempts were correct. For dermis, the success rate is 63.33% with 38 correct classifications. For fat, 60 out of 60 classification attempts were successful, which equals a success rate of 100.00%. Altogether, 70.56% of the individual classifications were correct. The classfied tissue types can now be visualized within the sensitive volumes enclosing the tissue volumes, in which the identified tissue types are predominant. Examplarily, locally visualized tissue types are shown in Figure 6.

### 3.2. Global Visualization

Figure 8 displays the classification success rate in a confusion matrix. As can be seen, the success rate has decreased in contrast to the local visualization. Less than one-third of the blood and skin phantom punctures were correctly classified. Approximately half of the classification attempts in fat were correct. It was also observed that the classification rate decreased with the number of repetitions.

We can now also consider the needle positions in which the electrodes are not completely located in one type of tissue phantom, i.e., the positions in which we are transitioning between two types of tissue phantom.

**Figure 9:**
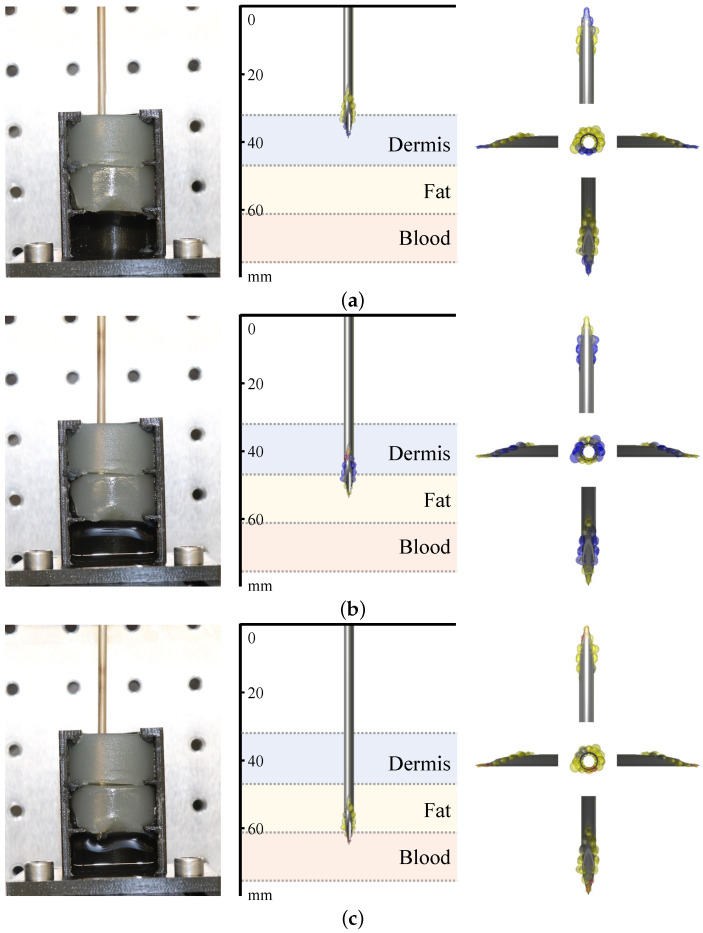
Global visualization experiments. The images on the left indicate the current position of the needle. The images in the center reveal the puncture depth and show the tissue types identified around the needle tip. The actual tissue types and their depths are displayed in the background. The images on the right provide different views of the needle. (**a**) The needle is transitioning from air to dermis. The system is falsely identifying fat rearmost and dermis foremost. (**b**) The needle is transitioning from dermis to fat. The system is correctly identifying dermis rearmost and fat foremost. (**c**) The needle is transitioning from fat to blood. The system is correctly identifying fat rearmost and blood foremost.

Based on the different colors in the visualization, we can determine when the transitions between tissue types happen. This can be seen in Figure 9. Whereas rear electrodes detect the already penetrated tissue type, the foremost electrodes already indicate a new tissue type. The spatial resolution of this needle is determined by the distances between the pairs of electrodes, which in this model is 2 mm in the longitudinal direction of the needle. For tissues with homogeneous properties, the spatial resolution is determined solely by the geometric arrangement of the electrodes. The puncture depth in the tissue being punctured is also identified through the queried position of the linear stage, which is displayed in the GUI.

## 4. Discussion

Impedance measurements by means of a needle equipped with stainless steel wire electrodes as well as tissue classification are possible with the proposed setup. Furthermore, using sensitive volumes for visualization that enclose the identified tissue types has demonstrated their functionality. In Figure 7, it can be seen that the sensitive volumes are distributed almost axisymmetrically around the needle. This is an indicator of the validity of this method, since the spatial sensitivity distribution itself is a quantity that depends solely on geometry, and we are using an axissymmetric needle geometry. The fluctuations that nevertheless remain can be attributed to numerical inaccuracies in the simulation and the fact that the mesh for the evaluation is not axisymmetric.

The hardware developed entails further uncertainties. The quality of the electrode that has been galvanized with gold cannot be guaranteed. Polarization effects can be more present when the gold layer has detached due to mechanical stress during the insertions. The fabrication process is performed completely manually, resulting in needles with different qualities and properties. In Figure 9, it can be seen that during the transitions, the visualization still displays the previous tissue layer for electrodes that should already be in the next tissue layer. This can also be accounted to the fabrication process, as the electrodes are not exactly positioned as in the ideal CAD needle model. As can be seen in Figure 1, the distal electrodes have a larger distance to the needle tip as designed. Compared to the CAD model, the distal electrodes are approximately 3–4 mm further away from the tip as the software expects, which is also noticeable in the visualization.

We also chose a relatively large diameter (14 G) to include more electrodes on the needle. It is questionable if this needle size would be used for venipuncture. In our current setup, the ability to use the needle as a cannula is not given anymore because it is plugged into the Luer–Slip connector of the adapter PCB. This has to be restored in future iterations. As our switching frequency between the excitation states is only 7 Hz, a slower relais board could be used instead of the multiplexers, eliminating any parasitic effect the switching electronics might impose.

Currently, the classification algorithm uses the unprocessed measurement data as a basis. The quality of the data can still be improved. The available data set had coefficients of variation for the real part between 0.735 (blood) and 0.941 (dermis) and for the imaginary part between 1.643 (fat) and 4.578 (dermis). Reducing the extent of variability would increase the classification success rate. It has not yet been investigated if preprocessing of the data or a different presentation of the data improves the success rate of the k-NN algorithm used. A coordinate transformation or feature scaling of the data could be investigated. In addition, a comparison of different classification algorithms such as support vector machines, or modifications of the k-NN algorithm, e.g., fixed-radius nearest neighbor, could be helpful.

It is obvious that the classification provided better results for single tissue phantoms, i.e., local visualization, than for the layered tissue structure (global visualization). This could be due to the fact that we used distilled water to rinse the needle prior to an insertion. However, it cannot be guaranteed that the distilled water has fully dried or evaporated before the measurement. Supporting this suspicion is the fact that we observed an improved classification success rate for needles that have been exposed to air for a longer time. Tissue parts from previous tissue types could also have remained at the exposed wire electrodes, which were than also dragged into the next tissue type, affecting the measurement result. In addition, we observed a slight leakage of blood phantom into the puncture holes once the layer above has been punctured, which also influences the measurement. The observation that the classifications became worse the higher the repetition is supporting this assumption. It would be advisable to perform the insertions with fresh tissue phantoms without any previous puncture holes. The classification rate of the fat phantom was highest for both test modes. This is probably because the recorded impedance data for dermis and blood are quite similar (cf. Figure 5). The data for fat differ more, which makes it better distinguishable.

## 5. Conclusions

For the first time, the concept of needle-based electrical impedance imaging has been presented. It exploits the sensitivity distribution of the electrode configuration in use. A prior determination of the sensitivity distribution is therefore pivotal. An average classification success rate of 70.56% for individual tissue phantoms was achieved. Future works will especially address hardware improvements and improvements to the classification process as well as the underlying dataset. Furthermore, a thin film-based approach on electrode structuring could be worth investigating as minimal structure sizes, and thus the spatial resolution, can be scaled down to the micrometer range. An implementation in C++ or C# is conveivable to avoid potential latency issues. In addition, more tissue types will be included to include more applications such as epidural anesthesia. Eventually, usability tests will be performed to evaluate the efficacy of the needle navigation system as an alternative to conventional imaging techniques.

## Figures and Tables

**Table 1:** Ingredients used for phantom fabrication.

Tissue Type	Ingredients (% *w*/*w*)					
	Distilled Water	Sodium Chloride	Agar	Gelatine	Propylene Glycol	5 M NaCl Solution
Skin	75.40	0.30	3.77	1.88	18.72	-
Fat	95.24	-	4.76	-	-	-
Blood	-	-	-	-	70.00	30.00

**Table 2:** Material parameters used for the simulation.

Entity	Material	σ (S/m)	$\epsilon_{r}$
Needle	Stainless Steel 410 Annealed	$1.74\times 10^{4}$	1
Insulation	PVDF	$6.77\times 10^{-13}$	8.1
Wires	Gold	$4.5\times 10^{7}$	1
Tissue	Blood	0.7	5120.02
	Fat	$4.34\times 10^{-2}$	101.49
	Dermis	0.25	$1.25\times 10^{4}$

## Data Availability

Data is contained within the article.

## Abbreviations

The following abbreviations are used in this manuscript:

AC/DC	Alternating Current/Direct Current
ASCII	American Standard Code for Information Interchange
CAD	Computer-Aided Design
CC	Current-Carrying
COM	Communication Port
EC	Electric Current
EMG	Electromyography
FEM	Finite Element Method
G	Gauge
GUI	Graphical User Interface
IA	Impedance Analyzer
IDE	Integrated Development Environment
k-NN	k-Nearest Neighbors
MDPI	Multidisciplinary Digital Publishing Institute
ML	Machine-Learning
PCB	Printed Circuit Board
PIVC	Peripheral Intravenous Catheter
PU	Pick-Up
PVDF	Polyvinylidene Difluoride
TCP/IP	Transmission Control Protocol/Internet Protocol
